# Dihydrocaffeic Acid Prevents UVB-Induced Oxidative Stress Leading to the Inhibition of Apoptosis and MMP-1 Expression via p38 Signaling Pathway

**DOI:** 10.1155/2019/2419096

**Published:** 2019-01-17

**Authors:** Mariana M. Oliveira, Bianca A. Ratti, Regina G. Daré, Sueli O. Silva, Maria da Conceição T. Truiti, Tânia Ueda-Nakamura, Rachel Auzély-Velty, Celso V. Nakamura

**Affiliations:** ^1^Programa de Pós-Graduação em Ciências Farmacêuticas, Universidade Estadual de Maringá, Maringá 87020-900, Brazil; ^2^Centre de Recherches sur les Macromolécules Végétales, Université Grenoble Alpes, Grenoble 38041, France

## Abstract

Chronic UVB exposure promotes oxidative stress, directly causes molecular damage, and induces aging-related signal transduction, leading to skin photoaging. Dihydrocaffeic acid (DHCA) is a phenolic compound with potential antioxidant capacity and is thus a promising compound for the prevention of UVB-induced skin photodamage. The aim of this study was to evaluate the antioxidant and protective effect of DHCA against oxidative stress, apoptosis, and matrix metalloproteinase (MMP) expression via the mitogen-activated protein kinase (MAPK) signaling pathway on L929 fibroblasts irradiated with UVB. DHCA exhibited high antioxidant capacity on 2,2-diphenyl-1-picrylhydrazyl (DPPH^•^), 2,2-azinobis-3-ethylbenzothiazoline-6-sulphonic acid (ABTS^•+^), and xanthine/luminol/xanthine oxidase (XOD) assays and reduced UVB-induced cell death in the neutral red assay. DHCA also modulated oxidative stress by decreasing intracellular reactive oxygen species (ROS) and extracellular hydrogen peroxide (H_2_O_2_) production, enhancing catalase (CAT) and superoxide dismutase (SOD) activities and reduced glutathione (GSH) levels. Hence, cellular damage was attenuated by DHCA, including lipid peroxidation, apoptosis/necrosis and its markers (loss of mitochondria membrane potential, DNA condensation, and cleaved caspase 9 expression), and MMP-1 expression. Furthermore, DHCA reduced the phosphorylation of MAPK p38. These findings suggest that DHCA can be used in the development of skin care products to prevent UVB-induced skin damage.

## 1. Introduction

Ultraviolet (UV) irradiation is one of the major exogenous harmful agents to the skin. This irradiation consists of UVC (100–280 nm), UVB (280–320 nm), and UVA (320–400 nm), but only UVB and UVA reach the earth's surface (95% UVA and 5% UVB). UVB penetrates the epidermis and the upper layer of the dermis, and despite representing the minor percentage of sunlight, it leads to greater skin damage than UVA at similar irradiation doses [1, 2]. UVB promotes oxidative stress by inducing exacerbated reactive oxygen species (ROS) production and decreasing endogenous antioxidants, such as catalase (CAT), superoxide dismutase (SOD), and reduced glutathione (GSH) [1, 3]. Oxidative stress could promote protein, mitochondrial, and DNA alterations as well as lipid peroxidation [4]. Moreover, high levels of ROS induce matrix metalloproteinase-1 (MMP-1), MMP-3, and MMP-9 expression by triggering the phosphorylation of mitogen-activated protein kinases (MAPKs) p38, JNK, and ERK. MMP-1 degrades collagen, while MMP-3 and MMP-9 also break down elastin, which together are the main structural proteins of the dermal extracellular matrix (ECM), and maintain the strength and elasticity of the skin [5]. In addition, MAPKs induced by oxidative stress could mediate apoptosis in skin cells [6]. These sets of skin alterations generated by the oxidative stress induced by chronic UVB exposure could contribute to photoaging development, which is characterized by deep wrinkling, loss of elasticity, dehydration, telangiectasia, and pigmentation alterations [4, 5].

In recent years, the amount of UV irradiation that reaches the earth's surface has been increasing due to the hole in the ozone layer, and sunscreens do not fully protect against the detrimental skin effects stimulated by UVB [3, 7]. Also, the average age of the world's population is still rising (World Population Ageing: 1950–2050, United Nations Population Division); thus, the skin becomes more susceptible to chronic damage promoted by UVB. Hence, further researches are needed to find complementary strategies to prevent skin photodamage.

**Figure 1:**
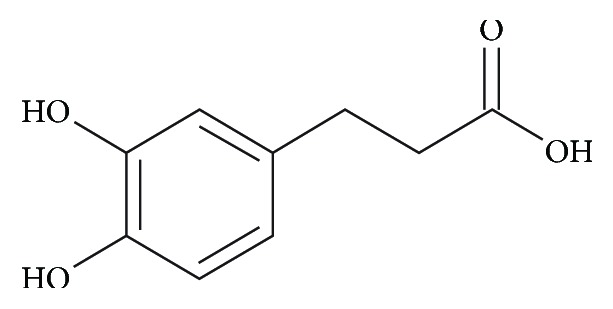
Chemical structure of DHCA.

Dihydrocaffeic acid (DHCA) (Figure 1) is a phenolic acid commonly found in the plasma and urine as a metabolite of several polyphenols of foods, beverages, and medicinal plants, such as chocolate, coffee, and wine extract [8]. It has also been isolated from plant species, including *Gynura bicolor* [9], *Nepeta teydea* [10], and *Selaginella stautoniana* [11]. The foregoing observations showed that DHCA presents *in vitro* antioxidant potential and anti-inflammatory and cytoprotective activities on keratinocytes irradiated with UV, decreases lipid peroxidation in human plasma and erythrocytes [8], and inhibits MMP-2 and MMP-9 in the brain tissue of rats [12]. We therefore sought to determine the molecular mechanisms involved in the protective and antiaging effects of DHCA on L929 fibroblasts irradiated with UVB.

## 2. Materials and Methods

### 2.1. Cell-Free Antioxidant Potential of DHCA

#### 2.1.1. ABTS Assay

The ABTS^•+^ (2,2-azinobis-3-ethylbenzothiazoline-6-sulphonic acid) scavenging ability of DHCA (Sigma-Aldrich, St. Louis, MO, USA) was evaluated by the ABTS assay [13]. To produce ABTS^•+^ radical cations, 7 mM ABTS stock solution was mixed with 2.45 mM potassium persulfate at room temperature in the dark for 16 h. The ABTS^•+^ solution was diluted with ethanol to obtain an absorbance of 0.70 (±0.05) at 734 nm (BioTek, PowerWave XS microplate spectrophotometer). Then, 7 *μ*L of DHCA (35 *μ*M) or quercetin (QT, 35 *μ*M) dissolved in ethanol was added to 200 *μ*L of the ABTS^•+^ solution. The absorbance was measured after 6 min at 25°C in the dark. The ABTS^•+^ scavenging ability of DHCA and QT was compared with a standard curve made with trolox (6-hydroxy-2,5,7,8-tetramethylchroman-2-carboxylic acid) solutions at different concentrations (5–250 *μ*g/mL); the results were expressed as *μ*M of trolox equivalent/*μ*M of sample.

#### 2.1.2. DPPH^•^ Assay

The DPPH^•^ (2,2-diphenyl-1-picrylhydrazyl) scavenging ability of DHCA was evaluated by mixing 100 *μ*L of DPPH^•^ methanol solution (65 *μ*M) with 100 *μ*L of DHCA or QT dissolved in methanol at different concentrations (2.7–16.5 *μ*M). The absorbance was measured at 517 nm (BioTek, PowerWave XS microplate spectrophotometer) after 30 min of incubation at 25°C in the dark. A negative control was produced with 100 *μ*L of methanol mixed in 100 *μ*L of DPPH^•^ and maintained under the same conditions as samples [14]. DPPH^•^ scavenging (%) was calculated using the following equation:

(1) $$\begin{array}{c} {DPPH}^{•}\;scavenging\;ability\left(\%\right)=\frac{{Abs}_{nc}-{Abs}_{s}}{{Abs}_{nc}}\times 100. \end{array}$$

Abs_nc_ is the absorbance of the negative control at 517 nm and Abs_s_ is the absorbance of the samples at 517 nm. The results were expressed as the IC_50_ values (concentration of sample that scavenges 50% of DPPH^•^).

#### 2.1.3. Xanthine/Luminol/Xanthine Oxidase (XOD) Assay

The superoxide anion (O_2_^•-^) scavenging capacity of DHCA was analyzed against O_2_^•-^ produced in a luminol-dependent chemiluminescent assay using the xanthine/luminol/XOD system [15]. To produce the reagent solution, 400 *μ*L of glycine buffer (0.1 M and 1 M of EDTA, pH 9.4) was mixed with 150 *μ*L of xanthine (6 mM), 10 *μ*L of luminol (0.6 mM), and 10 *μ*L of DHCA or QT (0.05–5.1 *μ*M) dissolved in ethanol (50%, *v*/*v*). To start the reaction, 100 *μ*L of fresh and cold XOD (20 mU/mL) was added to the reagent solution; one min later, the mixture was read on 96-well plates on a luminescence reader (Molecular Devices, SpectraMax L Microplate Reader). O_2_^•-^ scavenging (%) was calculated using the following equation:

(2) $$\begin{array}{c} {O_{2}}^{•‐}\;scavenging\;ability\left(\%\right)=\frac{{Cl}_{nc}-{Cl}_{s}}{{Cl}_{nc}}\times 100. \end{array}$$

Cl_nc_ is the chemiluminescence of the negative control and Cl_s_ is the chemiluminescence of the samples. The results were expressed as the IC_50_ values.

### 2.2. Effect of DHCA against UVB-Induced Cell Death

#### 2.2.1. Cell Culture

Fibroblast L929 cell line (NCTC clone 929 [L cell, L929, derivative of Strain L] (ATCC® CCL1™, Manassas, USA) was kindly provided by Dr. Maria José Vieira Fonseca (Faculty of Pharmaceutical Sciences of Ribeirão Preto, University of São Paulo, Ribeirão Preto, Brazil). L929 cells were maintained and cultured in Dulbecco's modified Eagle's medium (DMEM, Life Technologies/Gibco Laboratories, Grand Island, NY, USA) supplemented with 2 mM L-glutamine, 10% (*v*/*v*) fetal bovine serum (FBS, Life Technologies/Gibco Laboratories, Grand Island, NY, USA), penicillin (50 U/mL), and streptomycin (50 *μ*g/mL) at 37°C in a 5% CO_2_ atmosphere.

#### 2.2.2. Cellular Viability

The cytotoxicity of treated L929 cells was assessed using the neutral red assay [16]. The cultured cell suspension was dispensed into a 96-well plate (2.5 × 10^4^ cells/well) and incubated for 24 h. Postincubation, the media were replaced with serum-free DMEM containing different concentrations (7, 14, 21, 28, and 35 *μ*M) of DHCA with a maximum of 0.6% (*v*/*v*) DMSO or by serum-free DMEM containing 0.6% DMSO and incubated for 24 h. The monolayers were washed after incubation with phosphate-buffered saline (PBS), and 200 *μ*L of neutral red (Interlab, São Paulo, SP, BR) solution (40 *μ*g/mL) was added. The supernatant was removed after 3 h, and the cells were fixed with an aqueous solution of 2% (*v*/*v*) formaldehyde and 1% (*w*/*v*) calcium chloride, and then 200 *μ*L of a solution of 1% (*v*/*v*) acetic acid and 50% (*v*/*v*) ethanol was added. Absorbance was measured at 540 nm (BioTek, PowerWave XS microplate spectrophotometer) after 15 min at 25°C in the dark. The percentage of viable cells was calculated relative to untreated cells.

#### 2.2.3. Treatment of Cells and UVB Irradiation

After 24 h of culture, L929 cells were treated with DHCA or 1 mM N-acetyl cysteine (NAC, Sigma-Aldrich, St. Louis, MO, USA) (as a positive control) dissolved in serum-free DMEM and incubated for 1 h. Thereafter, media with treatment was replaced with Hank's balanced salt solution (HBSS, Sigma-Aldrich, St. Louis, MO, USA) and cells were irradiated with UVB using one UVB lamp (Philips, TL 40W/12 RS SLV) at a fixed distance of 20 cm from the surface of the cell culture plates. The UVB dose was 600 mJ/cm^2^, which demonstrated to cause 50% cell death in preliminary studies performed by our research group [17]. The irradiation intensity was measured by radiometer (Vilber Lourmat, VLX-3W), equipped with a sensor to detect UVB (detects peak of 312 nm) (Vilber Lourmat, CX-312). After irradiation, cells were kept in serum-free DMEM for 24 h and the analyses were performed.

To evaluate intracellular ROS and extracellular H_2_O_2_ production, lipid peroxidation, and mitochondrial membrane potential, L929 cells were seeded into black 96-well plates at a density of 2.5 × 10^4^ cells/well. For the cytoprotection assay, L929 cells were seeded into 24-well plate at a density of 1.25 × 10^5^ cells/well. For fluorescence microscopy assays (lipid peroxidation, mitochondrial membrane potential, Hoechst 33342, and apoptosis assays), L929 cells were seeded under glass discs into 24-well plates at a density of 1.25 × 10^5^ cells/well. For all other experiments, L929 cells were seeded into 6-well plates at a density of 8 × 10^5^ cells/well.

#### 2.2.4. Cytoprotection Assay

The capacity of DHCA to protect L929 cells against death induced by UVB was evaluated by the neutral red assay [16]. Cells were treated with different concentrations (7, 14, 21, 28, and 35 *μ*M) of DHCA, 1 mM NAC, or 0.6% DMSO dissolved in serum-free DMEM for 1 h and were irradiated with UVB (600 mJ/cm^2^). After 24 h, cells were subjected to the neutral red assay. The percentage of viable cells was calculated relative to untreated and nonirradiated cells. The concentration of DHCA (35 *μ*M) that significantly decreased UVB-induced cell death was chosen for further assays.

### 2.3. Effect of DHCA against UVB-Induced Oxidative Stress

#### 2.3.1. Intracellular ROS Measurement

The production of ROS induced by UVB was conducted using the fluorescent probe 2′,7′-dichlorodihydrofluorescein diacetate (H_2_DCFDA, Eugene, OR, USA). Cells were treated with 35 *μ*M DHCA or 1 mM NAC for 1 h and maintained with 10 *μ*M H_2_DCFDA for 45 min at 37°C. Afterwards, cells were irradiated with UVB (600 mJ/cm^2^) and fluorescence intensity was immediately measured in a microplate reader (PerkinElmer, Victor X-3) with *λ*_exc_ 488 nm and *λ*_em_ 525 nm [18]. Protein concentrations were quantified by the Bradford method using Bio-Rad protein assay reagent (Bio-Rad, CA, USA) and bovine serum albumin as the standard.

#### 2.3.2. Extracellular H_2_O_2_ Measurement

UVB-induced H_2_O_2_ was evaluated using the Amplex Red kit assay (Invitrogen, Eugene, OR, USA) according to the manufacturer's instructions. Briefly, immediately after irradiation, cells were incubated with 15 *μ*M Amplex Red and 0.15 U/mL horseradish peroxidase in 100 mM Tris-HCl pH 7.5 for 30 min at 25°C. Fluorescence intensity was quantified in a microplate reader (PerkinElmer, Victor X-3) with *λ*_exc_ 563 nm and *λ*_em_ 590 nm. The Bradford method was used to quantify protein concentrations.

#### 2.3.3. Cellular Antioxidant Defense Measurements

To verify cellular antioxidant defenses, catalase (CAT) and superoxide dismutase (SOD) activities and GSH levels were assessed. Then, 24 h after treatment and irradiation, cells were washed twice with PBS and scraped in cell lysis buffer (10 mM Tris-HCl, 1% Triton X-100, pH 7.4). Cellular lysate was obtained after sonication for 1 min (5 s on, 5 s off, 30% amplitude) on ice and centrifugation at 14000 rpm for 10 min at 4°C. Protein concentrations were determined by the Bradford method. The supernatants were used to evaluate CAT and SOD activities and GSH levels.

CAT activity was measured by monitoring H_2_O_2_ consumption at 240 nm as a result of the catalytic activity of CAT present in samples [19]. Then, 300 *μ*L of H_2_O_2_ (30 mM) was added to 700 *μ*L of potassium phosphate buffer (50 mM, pH 7.0) containing supernatant aliquots (50 *μ*g/mL of protein) at 25°C. The decomposition rate of H_2_O_2_ was assessed spectrophotometrically at 240 nm (Shimadzu, UV-1700) and the results were expressed as *μ*M/min/*μ*g protein.

To determine SOD activity, 930 *μ*L of buffer (200 mM Tris HCl, 2 mM EDTA, pH 8.2) containing supernatant aliquots (50 *μ*g/mL of protein) was mixed with 70 *μ*L of pyrogallol (15 mM) (Sigma-Aldrich, St. Louis, MO, USA). After 2 min of incubation at room temperature, the absorbance was assessed at 420 nm (Shimadzu, UV-1700) [20]. The amount of SOD that inhibited 50% of the pyrogallol oxidation compared to the negative control was considered one unit of SOD activity, and the results were expressed as unit of SOD/*μ*g protein.

GSH content was measured by mixing 180 *μ*L of buffer (100 mM sodium phosphate, 5 mM EDTA, pH 8.0) containing cellular supernatants (50 *μ*g/mL of protein) with 10 *μ*L of *ο*-phthalaldehyde (7.5 mM) (Sigma-Aldrich, St. Louis, MO, USA). After 15 min of incubation at room temperature, fluorescence intensity was quantified in a microplate reader (PerkinElmer, Victor X-3) with *λ*_exc_ 350 nm and *λ*_em_ 420 nm [21]. The capacity of DHCA to restore GSH levels was compared with a standard curve made with GSH (Sigma-Aldrich, St. Louis, MO, USA) solutions at different concentrations (31–1000 *μ*g/mL); the results were expressed as *μ*g GSH/*μ*g of protein.

### 2.4. Effect of DHCA against UVB-Induced Oxidative Stress Damage

#### 2.4.1. Lipid Peroxidation Assay

Lipid peroxidation induced by UVB was carried out using the fluorescent probe diphenyl-1-pyrenylphosphine (DPPP, Invitrogen, Eugene, OR, USA) [22]. After 24 h of irradiation, cells were incubated with 20 *μ*M DPPP for 30 min at 37°C. Fluorescence intensity was measured in microplate reader (PerkinElmer, Victor X-3) with *λ*_exc_ 351 nm and *λ*_em_ 380 nm and protein concentrations were quantified by the Bradford method. For fluorescence microscopy, cells were subjected to the same treatment as described above and then visualized on a fluorescence microscope (Olympus, BX51, 40x); pictures were captured using an Olympus UC30 camera.

#### 2.4.2. Mitochondrial Membrane Potential (Δ*ψ*_m_) Measurement

The Δ*ψ*_m_ was measured using the fluorescent dye rhodamine 123 (Rh 123, Sigma-Aldrich, St. Louis, MO, USA) [23]. After 24 h of irradiation, cells were washed with 0.9% (*w*/*v*) sodium chloride and incubated with 26.2 *μ*M Rh 123 for 15 min at 37°C. Fluorescence intensity was measured in a microplate reader (PerkinElmer, Victor X-3) with *λ*_exc_ 488 nm and *λ*_em_ 525 nm, and protein concentrations were determined by the Bradford method. For fluorescence microscopy, after the same treatment and irradiation as described above, cells were visualized on a fluorescence microscope and pictures were captured.

#### 2.4.3. Apoptosis Assay

Phosphatidylserine on the outer leaflet of apoptotic cells was detected using annexin V-fluorescein isothiocyanate (FITC) binding (Invitrogen, Eugene, OR, USA). After 24 h of irradiation, cells were dissociated in cell dissociation buffer enzyme-free PBS-based (Life Technologies/Gibco Laboratories, Grand Island, NY, USA), washed with HBSS 0.25% EDTA, resuspended in 100 *μ*L of binding buffer (140 mM NaCl, 5 mM CaCl_2_, 10 mM HEPES-Na, pH 7.4), followed by staining with 2 *μ*L annexin V-FITC for 15 min at room temperature. Then, 400 *μ*L of binding buffer was added and 10,000 events were acquired using a flow cytometer (Becton Dickinson, FACSCalibur). Data were analyzed using CellQuest software.

#### 2.4.4. Acridine Orange and Propidium Iodide Double Staining

To distinguish viable, late apoptotic, and necrotic cells, double staining with acridine orange (Sigma-Aldrich, St. Louis, MO, USA) and propidium iodide (Invitrogen, Eugene, OR, USA) was performed [24]. Following 24 h of irradiation, cells were washed twice with PBS and incubated with 1 *μ*g/mL acridine orange and 1 *μ*g/mL propidium iodide diluted in PBS for 10 min at room temperature in the dark. Cells were then photographed using a fluorescence microscope and pictures were captured. For each experiment, 100 cells were counted and classified as viable (green), late apoptotic (orange nuclei), or necrotic (red nuclei) cells.

#### 2.4.5. Nuclear Staining with Hoechst 33342

Condensed nuclei in apoptotic cells were evaluated by Hoechst 33342 assay according to the manufacturer's instructions. Afterward 24 h of irradiation, cells were stained with 8 *μ*M Hoechst 33342 (Invitrogen, Eugene, OR, USA) for 15 min at 37°C. Stained cells were visualized on a fluorescence microscope and pictures were captured. Condensed nuclei were quantitated into 100 cells on each experiment.

#### 2.4.6. Western Blot Analysis

Western blot analysis was performed to detect phosphorylated JNK and p38, MMP-1, MMP-3, MMP-9, and caspase 9. After 24 h of irradiation, cells were harvested and lysed with lysis buffer containing 100 mM Tris-HCl pH 7.4, 2% (*w*/*v*) SDS, 5% (*v*/*v*) 2-mercaptoethanol, and 30% (*v*/*v*) glycerine. 1% (*v*/*v*) protease inhibitor cocktail (Sigma-Aldrich, St. Louis, MO, USA) containing 104 mM AEBSF, 80 *μ*M aprotinin, 4 mM bestatin, 1.4 mM E-64, 2 mM leupeptin, and 1.5 mM pepstatin and 1% (*v*/*v*) phosphatase inhibitor cocktail (Sigma-Aldrich, St. Louis, MO, USA) containing sodium orthovanadate, sodium molybdate, sodium tartrate, and imidazole were also added in lysis buffer. The protein concentration of each sample was determined by the Bradford method. Equal amounts of proteins were electrophoresed on 12% SDS-PAGE, transferred to a nitrocellulose membrane (GE Healthcare, Little Chalfont, Buckinghamshire, UK), and blocked for 1 h in Tris-buffered saline with 0.1% (*v*/*v*) Tween 20 (TBST) containing 5% bovine serum albumin (BSA). The membranes were incubated overnight at 4°C with the primary antibody against JNK (sc-7345), phosphorylated JNK (sc-6254), p38 (sc-7972), phosphorylated p38 (sc-166182), MMP-1 (sc-21731), MMP-3 (sc-21732), MMP-9 (sc-393859), and caspase 9 (sc-56076) diluted 1 : 500 and *β*-actin (sc-69879) diluted 1 : 10000 (Santa Cruz Biotechnology, Santa Cruz, CA, USA) in TBST plus 3% BSA. The membranes were then washed with TBST and incubated for 2 h at room temperature with HRP-conjugated secondary antibody for anti-mouse diluted 1 : 10000 (Santa Cruz Biotechnology, Santa Cruz, CA, USA) in TBST plus 3% BSA. Protein bands were detected with western blotting luminol reagent (Santa Cruz Biotechnology, Santa Cruz, CA, USA) and visualized with the imaging system (Bio-Rad, ChemiDoc™ MP). The relative amounts of proteins associated with each antibody were normalized to the respective *β*-actin bands.

### 2.5. Statistical Analysis

Data are expressed as mean ± standard deviation (SD) of at least three independent experiments. The statistical significance of differences between different groups was evaluated by one-way analysis of variance (ANOVA) followed by the Tukey test using Prism 5.0 software. Values of *p* < 0.05 were considered statistically significant.

## 3. Results

### 3.1. Cell-Free Antioxidant Potential of DHCA

The radical scavenging capacity of DHCA was evaluated against three different radicals (Table 1). The results of DHCA were compared with values obtained for QT, a flavonoid with high antioxidant potential described in the literature. DHCA and QT showed similar antioxidant capacity in the ABTS^•+^ assay, while DHCA had higher capacity than QT in DPPH^•^ assay, resulting in a lower IC_50_ value. The O_2_^•-^ scavenging ability of DHCA was lower than the ability of QT in the XOD assay, with a higher IC_50_ value. However, DHCA showed similar or even better results than other natural products (DHCA IC_50_ 0.17 *μ*g/mL, *Byrsonima crassifolia* purified extract IC_50_ 0.23 *μ*g/mL; *Byrsonima crassifolia* fraction IC_50_ 0.10 *μ*g/mL) [25].

### 3.2. DHCA Protects L929 Cells from UVB-Induced Death

**Figure 2:**
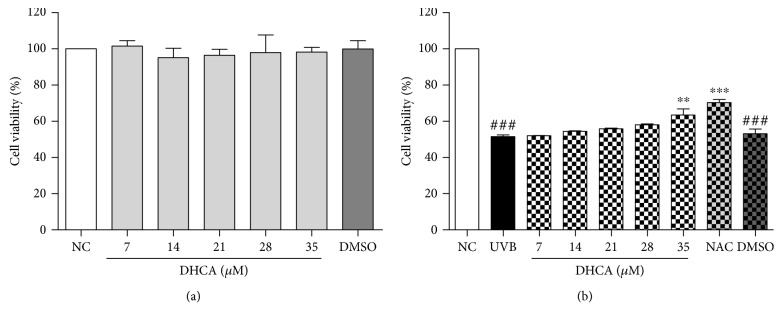
Cell viability using DHCA (7–35 *μ*M) and DMSO (0.6%) on L929 cells evaluated by the neutral red assay after 24 h of treatment (a). Protective effect of pretreatment with DHCA (7–35 *μ*M), 1 mM NAC (as positive control), and DMSO (0.6%) against UVB-induced L929 cell death evaluated by neutral red assay after 24 h of UVB exposure (600 mJ/cm^2^) (b). Each column represents the mean ± SD (*n* = 3). ^###^*p* < 0.001: significantly different from nonirradiated and nontreated cells (NC); ^∗∗^*p* < 0.01 and ^∗∗^*p* < 0.001: significantly different from irradiated and nontreated cells (UVB).

As shown by Figure 2(a), DHCA was not cytotoxic to L929 cells in all concentrations evaluated (7–35 *μ*M) in the neutral red assay, with a cell viability of 98.3% at the highest concentration. After L929 cells were exposed to UVB (600 mJ/cm^2^) (Figure 2(b)), cell viability decreased to 51.8% compared to nontreated and nonirradiated cells (NC). The treatment with DHCA attenuated UVB-induced cell death in a dose-dependent manner. The highest concentration of DHCA (35 *μ*M) significantly decreased cell death (63.4% of cell viability). Thus, this concentration was chosen to perform the next analyses. Similarly, 1 mM NAC, a well-known antioxidant, inhibited UVB-induced cell death (70.4% of cell viability). As expected, DMSO alone was not cytotoxic to L929 cells (99.9% of cell viability) and did not decrease the cell death induced by UVB (53.1% of cell viability).

### 3.3. Antioxidant Potential of DHCA on L929 Cells

**Figure 3:**
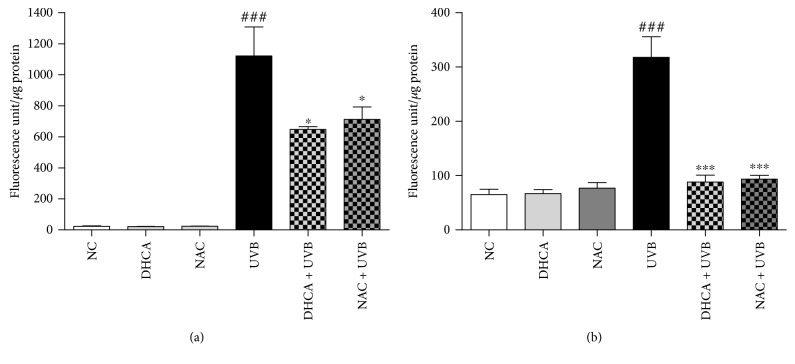
DHCA reduces UVB-induced ROS. L929 cells were treated with 35 *μ*M DHCA or 1 mM NAC for 1 h, irradiated with UVB (600 mJ/cm^2^), and immediately after irradiation, intracellular ROS (a) and extracellular H_2_O_2_ (b) production were evaluated by H_2_DCFDA and Amplex Red kit assay, respectively. Each column represents the mean ± SD (*n* = 3). ^###^*p* < 0.001: significantly different from nonirradiated and nontreated cells (NC); ^∗^*p* < 0.05 and ^∗∗∗^*p* < 0.001: significantly different from irradiated and nontreated cells (UVB).

To verify if the antioxidant capacity of DHCA contributes to protection against UVB-induced cell death, we evaluated intracellular ROS and extracellular H_2_O_2_ production. The capacity of DHCA to reduce ROS generation induced by UVB on L929 cells was evaluated using H_2_DCFDA assay (Figure 3(a)). H_2_DCFDA is a nonfluorescent probe that is deesterified by intracellular esterases and is oxidized by intracellular ROS to fluorescent 2′,7′-dichlorofluorescein [18]. DHCA and NAC alone did not alter the fluorescence intensity compared to NC. UVB significantly increased ROS production compared to NC. Pretreatment with 35 *μ*M DHCA reduced ROS production by 39.1% compared to irradiated and nontreated cells (UVB). The capacity of DHCA to inhibit UVB-induced ROS production was slightly higher than 1 mM NAC (inhibition of 34.9%).

To more specifically quantify H_2_O_2_ production, the Amplex Red kit assay was used. Amplex Red is oxidized to fluorescent resorufin by H_2_O_2_ in a reaction catalyzed by HRP. In the same way, DHCA and NAC alone did not increase H_2_O_2_ levels (Figure 3(b)). As expected, UVB-irradiated cells stimulated H_2_O_2_ production compared to NC. Pretreatment with 35 *μ*M DHCA and 1 mM NAC suppressed the H_2_O_2_ production induced by UVB to almost NC levels (72.2% and 69.2% inhibition compared to UVB, respectively).

### 3.4. DHCA Restores Endogenous Antioxidants

**Figure 4:**
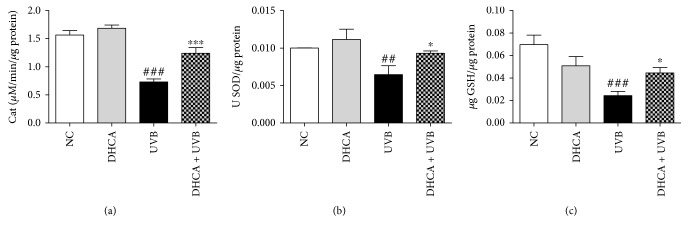
Capacity of DHCA to decrease antioxidant defense depletion induced by UVB. L929 cells were treated with 35 *μ*M DHCA for 1 h, irradiated with UVB (600 mJ/cm^2^), and after 24 h, CAT (a) and SOD (b) activities and GSH levels (c) were evaluated by H_2_O_2_ consumption at 240 nm, pyrogallol oxidation and *ο*-phthalaldehyde assays, respectively. Each column represents the mean ± SD (*n* = 3). ^###^*p* < 0.001 and ^##^*p* < 0.01: significantly different from nonirradiated and nontreated cells (NC); ^∗^*p* < 0.05 and ^∗∗∗^*p* < 0.001: significantly different from irradiated and nontreated cells (UVB).

It is well described on the literature that UVB diminishes endogenous antioxidants, contributing to oxidative stress development [1, 3]. Our results confirm that UVB irradiation significantly decreased endogenous antioxidant defenses by decreasing CAT and SOD activities by 53.1% and 35.5%, respectively, and depleting GSH levels by 64.3% compared to NC (Figure 4). Pretreatment with 35 *μ*M DHCA partially reversed these alterations by increasing CAT and SOD activities by 32.5% and 28.5%, respectively, and restoring 28.4% of the GSH levels compared to UVB.

### 3.5. DHCA Reduces UVB-Stimulated Oxidative Cellular Damages

#### 3.5.1. Lipid Peroxidation

**Figure 5:**
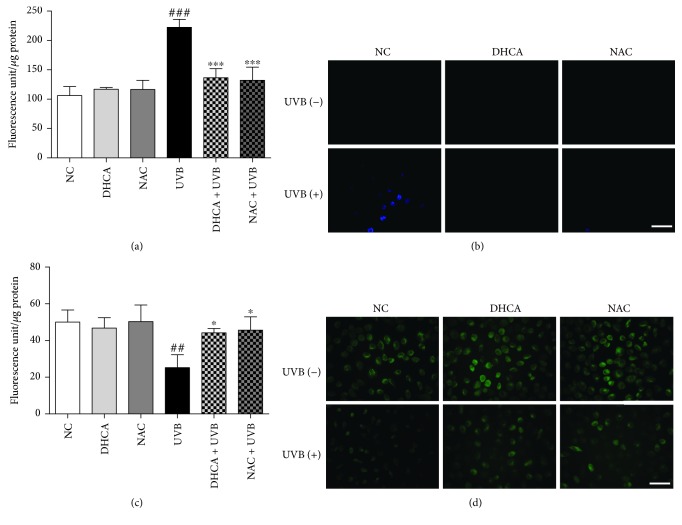
DHCA attenuates UVB-induced lipid peroxidation and loss of Δ*ψ*_m_. L929 cells were treated with 35 *μ*M DHCA or 1 mM NAC for 1 h and irradiated with UVB (600 mJ/cm^2^). After 24 h, cells were stained with DPPP or Rh 123 to evaluate lipid peroxidation and Δ*ψ*_m_ by fluorimetry (a, c) and fluorescence microscopy (b, d), respectively. Each column represents the mean ± SD (*n* = 3). ^###^*p* < 0.001 and ^##^*p* < 0.01: significantly different from nonirradiated and nontreated cells (NC); ^∗∗∗^*p* < 0.001 and ^∗^*p* < 0.05: significantly different from irradiated and nontreated cells (UVB). Scale bars: 50 *μ*m.

Lipid peroxidation is known to be associated with oxidative stress induced by UVB [26]. The antilipid peroxidation capacity of DHCA was assessed using the DPPP stain, which reacts stoichiometrically with lipid hydroperoxides to produce fluorescent DPPP oxide [22]. The fluorimetry analysis indicates that lipid peroxidation was two times higher in UVB than in the NC or in cells only treated with DHCA or NAC (Figure 5(a)). Both DHCA (35 *μ*M) and the standard antioxidant NAC (1 mM) inhibited the production of lipid hydroperoxides (38.3% and 40.2% of inhibition, respectively). The fluorescence microscopy images were in agreement with the results observed on fluorimetric analysis (Figure 5(b)).

#### 3.5.2. Δ*ψ*_m_

To investigate Δ*ψ*_m_ alterations in UVB-irradiated cells, Rh123 stain was used. Rh 123 is a lipophilic cationic dye that distributes according to the negative membrane potential across the mitochondrial inner membrane and emits fluorescence [23]. Hence, a loss of fluorescence intensity indicates a decrease in Δ*ψ*_m_. The fluorimetric analysis showed that the exposure of cells to UVB leads to a significant decrease in fluorescence intensity (50.1% of Δ*ψ*_m_ loss) compared to the NC or cells only pretreated with DHCA or NAC (Figure 5(c)). On the other hand, the treatment with 35 *μ*M DHCA or 1 mM NAC before UVB exposure prevented the loss of Δ*ψ*_m_ (10.6% and 7.9% of Δ*ψ*_m_ loss, respectively). The fluorescence microscopy confirms the reduced fluorescence intensity on irradiated L929 cells, as well as the protection conferred by the DHCA (Figure 5(d)).

#### 3.5.3. Assessment of Viability, Apoptosis, and Necrosis

**Figure 6:**
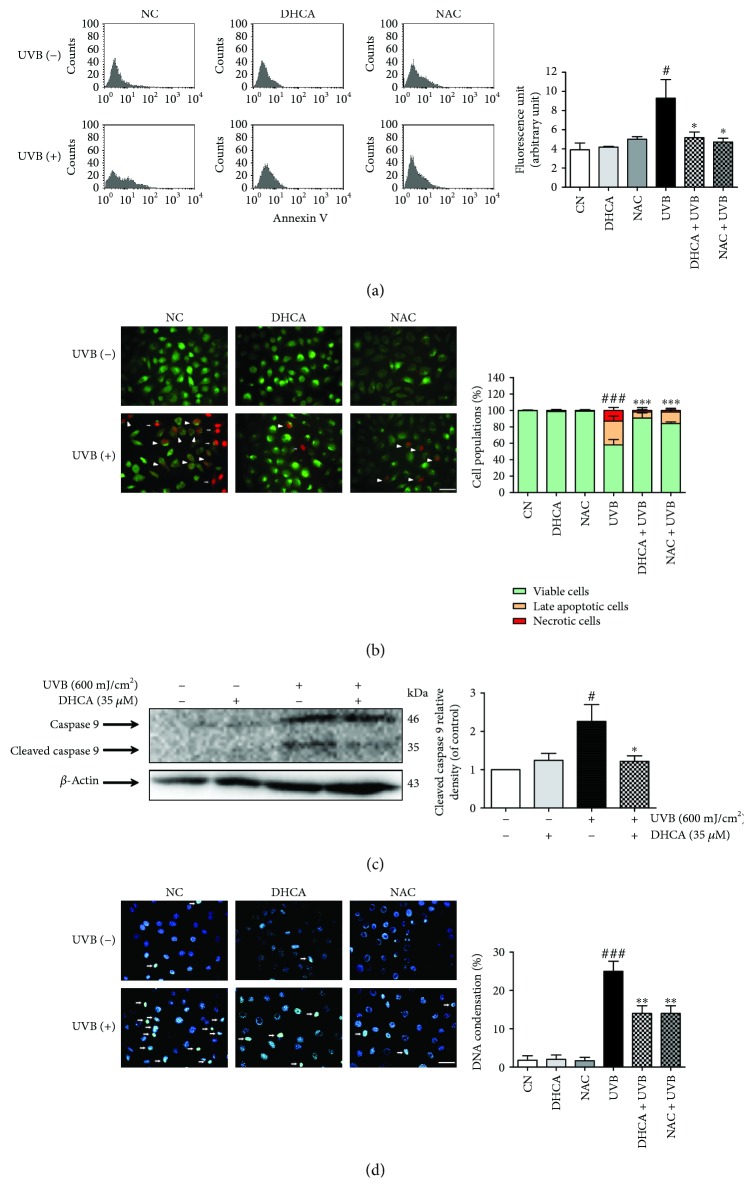
Capacity of DHCA to decrease apoptosis features induced by UVB. L929 fibroblasts were pretreated with 35 *μ*M DHCA or 1 mM NAC for 1 h, irradiated with UVB (600 mJ/cm^2^), and after 24 h, analyses were performed. (a) Cells were stained with annexin V, and apoptotic cells were quantified by flow cytometry. (b) Cells were double stained with acridine orange and propidium iodide, and viable cells (green fluorescence nuclei), late apoptotic cells (orange fluorescence nuclei, white headless arrow), and necrotic cells (red fluorescence nuclei, white arrows) were observed on fluorescence microscopy and quantitated. (c) Active caspase 9 expression was determined and quantified by western blot analysis using specific antibodies. (d) Cells were stained with Hoechst 33342 stain, and cells with condensed nuclei (white arrows) were observed on fluorescence microscopy and quantitated. Each column represents the mean ± SD (*n* = 3). ^###^*p* < 0.001 and ^#^*p* < 0.05: significantly different from nonirradiated and nontreated cells (NC); ^∗∗∗^*p* < 0.001, ^∗∗^*p* < 0.01, and ^∗^*p* < 0.05: significantly different from irradiated and nontreated cells (UVB). Scale bars: 50 *μ*m.

The effect of DHCA against apoptosis induced by UVB was firstly determined by annexin V staining (Figure 6(a)). Under apoptosis, phosphatidylserine becomes exposed on the outside leaflet of the membrane; thus, the detection of phosphatidylserine with annexin V allows the estimation of apoptosis [27]. UVB irradiation increased apoptosis by 42.5% compared to NC, and pretreatment with DHCA and NAC significantly attenuated apoptosis (41.6% and 46.7% of inhibition compared to UVB, respectively).

In order to differentiate viable, late apoptotic, and necrotic cells, we performed acridine orange and propidium iodide double staining. These markers are intercalating nucleic acid fluorochromes. Acridine orange permeates the membranes of viable cells and emits green fluorescence when intercalated with nucleic acids. Propidium iodide only permeates cells with a loss of membrane integrity and emits red fluorescence when it intercalates with nucleic acids. Then, when cells are double-marked with both fluorochromes, late apoptotic cells (nuclei emit orange fluorescence) or necrotic cells (red nuclei due to highest intensity emission of PI) can be revealed [28].

As shown by Figure 6(b), in the NC and cells treated with 35 *μ*M DHCA and 1 mM NAC alone, only viable cells with green fluorescent nuclei were observed (99.7%, 98.6% and 99% of cells with green fluorescent nuclei, respectively). UVB irradiation induces apoptosis (29% of cells with orange fluorescent nuclei) and also leads to necrosis (13% of cells with red fluorescent nuclei). However, the treatment with 35 *μ*M DHCA and 1 mM NAC significantly decreased apoptosis (11% and 13% of cells with orange fluorescent nuclei, respectively) and necrosis (2% and 3% of cells with red fluorescent nuclei, respectively).

Another parameter that reveals apoptosis is caspase 9 cleavage, indicating activation of the intrinsic apoptotic signaling pathway [27]. The levels of cleaved caspase 9 expression were 2.3-fold higher in fibroblasts exposed to UVB than in the NC. The cells pretreated with DHCA exhibited a reduction in UVB-induced cleaved caspase 9 expression, which was just 1.2-fold higher than the NC (Figure 6(c)). These results indicate that the intrinsic pathway is involved in UVB-induced apoptosis on L929 cells at the described conditions.

#### 3.5.4. Nuclear Condensation

Hoechst 33342 is a nucleic acid stain that emits blue fluorescence when bound to dsDNA and can distinguish intact nuclei in viable cells from condensed nuclei in apoptotic cells. Normal nuclei were found in NC and in cells treated with 35 *μ*M DHCA or 1 mM NAC alone (1% of condensed nuclei for NC and 2% of condensed nuclei for cells treated with DHCA and NAC), whereas UVB irradiation significantly increased condensed nuclei (25%) (Figure 6(d)). As expected, pretreatment with 35 *μ*M DHCA and 1 mM NAC reduced nuclei condensation induced by UVB (14%, both treatments).

### 3.6. DHCA Attenuates MMP Expression

**Figure 7:**
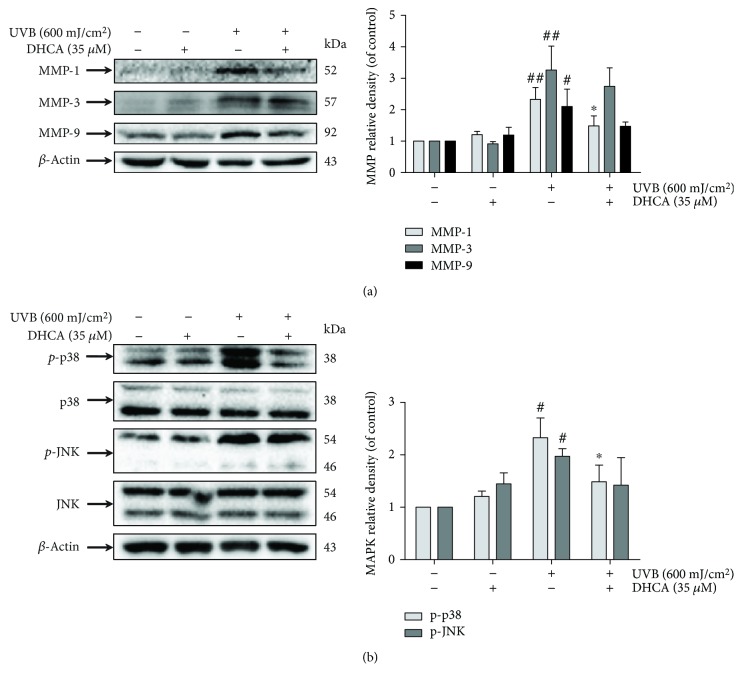
Effect of DHCA on UVB-induced MMP expression (a) and the MAPK signaling pathway (b). L929 cells were treated with 35 *μ*M DHCA for 1 h, irradiated with UVB (600 mJ/cm^2^), and protein expression was evaluated after 24 h by western blot assay using specific antibodies. Each column represents the mean ± SD (*n* = 3). ^##^*p* < 0.01 and ^#^*p* < 0.05: significantly different from nonirradiated and nontreated cells (NC); ^∗^*p* < 0.05: significantly different from irradiated and nontreated cells (UVB).

Dermal ECM is mainly composed of collagen and elastin fibers and is fundamental to maintaining healthy skin [29]. In order to assess the way in which DHCA prevents ECM degradation induced by UVB, the inhibitory effect of DHCA on MMPs expression was further investigated. As shown in Figure 7(a), the expression of MMP-1, MMP-3, and MMP-9 was significantly increased when L929 cells were irradiated with 600 mJ/cm^2^ of UVB (2.3-fold, 3.3-fold, and 2.1-fold compared to the NC, respectively). However, treatment with 35 *μ*M DHCA before UVB exposure significantly attenuated the expression of MMP-1 (1.5-fold compared to the NC) and slightly attenuated MMP-3 and MMP-9 expression (2.7-fold and 1.5-fold compared to the NC, respectively).

### 3.7. DHCA Inhibits JNK and p38 MAPK Phosphorylation

The MAPK signaling pathway is activated by large amounts of ROS produced in cells irradiated with UVB, followed by downstream transcription factor phosphorylation, leading to the expression of apoptotic factors and MMPs [5, 30]. Since DHCA reduces apoptosis and MMP expression, we intended to verify whether DHCA may act by inhibiting phosphorylation of the MAPK subfamilies JNK and p38 (Figure 7(b)). The phosphorylation of JNK and p38 was increased after UVB exposure (2.0-fold and 2.4-fold compared to NC, respectively), whereas pretreatment with 35 *μ*M DHCA significantly diminished the phosphorylation of p38 (1.3-fold compared to NC) and JNK to a lower extent (1.4-fold compared to the NC).

## 4. Discussion

Human skin provides the main protection barrier against environmental agents, as well as conferring external beauty. Extrinsic skin aging, also referred as to skin photoaging, occurs due to the cumulative exposure to external stimuli, and UVA and UVB irradiation is one of the most harmful factors [31]. UVB promotes oxidative stress by increasing ROS production and decreasing endogenous antioxidants, directly causing molecular damage and activating aging-related signal transduction. In addition to photoaging, the alterations induced by UVB also can lead to other skin disorders, including skin cancer [5, 31].

Dermal ECM determines the structural and mechanical properties of the skin, which is essential to maintain a good skin appearance. The primary dermis cells responsible for producing ECM components are fibroblasts. These cells scarcely proliferate and are therefore susceptible to continuous UVB-induced damage accumulation [32]. Many studies have demonstrated that fibroblasts in monolayer culture reproduce various forms of UVB-induced photodamage at the cellular level [1, 17]. To prevent damage generated by UVB irradiation and maintain healthy and beautiful skin, several studies have focused on compounds or extracts with antioxidant properties [17, 25]. It has been reported that DHCA exhibits antioxidant activity, which is attributed to catechol structure and carboxyl group found in DHCA [33]. Therefore, we studied the protective effects of DHCA against UVB photodamage on L929 fibroblasts. NAC was used as an antioxidant standard in some analyses due to its ROS scavenging capacity and ability to protect against skin photodamage reported in the literature [34].

First we showed, in a cell-free system, the antioxidant potential of DHCA by ABTS^•+^, DPPH^•^, and XOD assays. These assays evaluate the ability of a compound to transfer an electron to ABTS^•+^, to donate an electron or hydrogen to DPPH^•^, and to scavenge O_2_^•-^, respectively. Our results showed that DHCA presents high antiradical capacity in all of the methodologies evaluated, confirming their previously reported antioxidant potential [8].

We also found that pretreatment with DHCA led to a significant reduction of UVB-induced fibroblast death and the antioxidant capacity of DHCA contributes to this protection. The antioxidant activity of DHCA in a cell system was evidenced by the decrease in UVB-induced ROS production, and particularly diminished H_2_O_2_ levels, to almost basal levels. Previous studies show that H_2_O_2_ leads to several forms of cellular damage, including protein oxidation and intrinsic apoptosis induction through the MAPK signaling pathway. Moreover, H_2_O_2_ can be converted into harmful compounds, including hypochlorous acid (HOCl) by the myeloperoxidase enzyme and the hydroxyl radical (HO^•^) by the Fenton reaction [5, 35].

To protect the organism against ROS overproduction and maintain homeostasis, mammalian cells contain an antioxidant system composed of enzymatic and nonenzymatic antioxidants. The SOD enzyme converts O_2_^•-^ into H_2_O_2,_ which is decomposed into H_2_O and O_2_ by the CAT enzyme [5]. GSH is the most abundant nonenzymatic antioxidant in the organism, which acts to scavenge ROS, regenerate other antioxidants, and is a cofactor for glutathione peroxidase [36]. However, as well as excessive UVB irradiation exposure increasing ROS generation, it also depletes the endogenous antioxidant system [1, 3]. Thus, we demonstrated that pretreatment with DHCA attenuated these alterations, increasing the activity of CAT and the SOD and GSH levels.

The cellular redox imbalance induced by UVB radiation might induce cellular damage [5]. The lipids of membranes are potential targets of ROS. Hydrogen atoms of polyunsaturated fatty acids are removed by ROS, and then peroxyl radicals are produced through the reaction between the extra electrons of lipids and molecular oxygen. Peroxyl radicals react with other lipids and produce other radicals, such as lipid hydroperoxides [26]. These cascade reactions damage cell membranes, which has a fundamental importance to cells, since it is a barrier against the extracellular environment, and might lead to cell death [37]. As expected, we showed that DHCA reduced oxidative stress cellular damage by decreasing lipid oxidative damage in the same way as the standard antioxidant NAC.

The oxidative stress induced by UVB is also involved in the activation of the intrinsic apoptotic signaling pathway [30]. Our data showed that excessive ROS levels, especially H_2_O_2_, associated with a decrease of the endogenous antioxidant system induced by UVB on L929 cells, caused the loss of Δ*ψ*_m,_ and significantly activated caspase 9, as observed by western blotting assays. As a result, these sets of alterations undergo apoptosis by the intrinsic apoptotic pathway, and, to a lesser extent, necrosis, analyzed by the annexin V assay and acridine orange and propidium iodide double staining assay. All of this damage was partially reversed by pretreatment with 35 *μ*M DHCA and in a similar way by 1 mM NAC. The intrinsic apoptotic signaling pathway induced by the excessive production of ROS is well described and is responsible for opening pores in the mitochondrial membrane, leading to changes in Δ*ψ*_m_. The giant pores are permeable to ions such as K^+^ and Cl^+^, and certain proteins which maintain the osmotic balance between the mitochondrial matrix and the perimitochondrial space. Then, the high concentration of proteins in the mitochondrial matrix induces the inflow of water to try diluting the matrix mitochondrial content. Consequently, the matrix swells, the inner membrane wrinkles are smoothed, and the outer membrane bursts. Thereafter, proteins are released from the cytosol, such as cytochrome *c*, which together with cytoplasmic apoptotic protease activating factor 1 (APAF-1) constituted the apoptosome, which activates caspase 9. Later, it activates the effector caspases 3 and 7 and conducted the cell to apoptosis [27].

The condensation of nuclei and its further fragmentation and rupture of DNA into strands are also characteristics of apoptosis. After the activation of effector caspases, DNase is activated, at the same time as the DNA repair proteins are inactivated or destructed, such as PARP-poly(ADP-ribose), leading to chromatin disintegration and fragmentation of DNA into 200 nucleotide fragments [27]. Here we showed that UVB induces nuclear condensation on L929 cells, evidenced by Hoechst 33342 method, while treatment with DHCA clearly suppressed it.

UVB irradiation can lead to different apoptotic responses according to doses and cell type used. It has been shown that at low and intermediate doses in keratinocytes, UVB induces early and late apoptosis, respectively. On the other hand, at the highest doses, UVB simultaneously activates both apoptotic and necrotic pathways [38]. Liu et al. [39] demonstrated that UVB at 90, 180, and 270 mJ/cm^2^ induces only apoptosis on L929 fibroblasts. Our results showed that UVB irradiation at higher doses (600 mJ/cm^2^) induces both apoptosis and necrosis on L929 fibroblasts, which suggests that UVB, at different doses, also generates distinct cell death responses in L929 fibroblasts.

In adult skin, the ECM comprises collagen fibers (70–80% of the skin dry weigh), mostly collagen type I (80% of all collagens), elastin fibers, proteoglycans, glycosaminoglycans, and water [29]. UVB-stimulated ROS increase MMP expression on keratinocytes and fibroblasts and trigger collagen and elastin degradation of ECM. MMP-1 (collagenase) breaks down intact type I, II, and III fibrillar collagens, whereas MMP-3 (stromelysin) and MMP-9 (gelatinase) degrade denatured collagen and elastin fibers [5, 40]. Additionally, tissue inhibitor of MMP (TIMP) expression is not increased or it is even decreased by UVB exposition. These set of alterations lead to ECM degradation and the appearance of photoaging signals [41]. It is well known that antioxidant extracts or compounds that inhibit MMP expression can be used to prevent skin aging [31]. Previous research has already shown that DHCA decreases MMP-2 and MMP-9 inhibition in the brain tissue of rats [12]. Herein, we showed that DHCA significantly attenuated UVB-induced MMP-1 expression, the major enzyme that degrades collagen. On the other hand, DHCA did not show a significant reduction in the expression of MMP-3 and MMP-9.

The MAPK signaling pathway is responsible for transmitting extracellular stimuli into the cell nucleus, which play crucial roles in many activities of cells, including apoptosis and MMP expression [6, 40]. UVB-induced ROS have been reported to contribute to the activation of both the JNK and p38 MAPK subfamilies on skin cells [5, 30]. p-JNK and p-p38 induce the release of mitochondrial cytochrome *c*, followed by the activation of caspase 9 and the effector caspases 3 and 7, resulting in apoptosis through the intrinsic pathway [30, 42]. Moreover, several studies have shown that p-JNK and p-p38 trigger MMP expression in fibroblasts after UVB exposure by activating the transcription factor AP-1 [31, 40]. Our results demonstrated that UVB phosphorylates JNK and p38 MAPKs in L929 cells, and DHCA attenuates the phosphorylation of both MAPK subfamilies, with a marked decrease mainly in p38.

Besides MAPK, other signaling pathways are involved in UVB-induced oxidative stress, among them the nuclear factor-kappa beta (NF-*κβ*) and the nuclear factor (erythroid-derived 2)-like 2 (Nrf2) [43]. UVB releases NF-*κβ* from its inhibitor (I*κ*B*α*), leading to the translocation of active NF-*κβ* to the nucleus. It results in activation of inflammatory cytokines and prostaglandins [44]. Nrf2 is a transcription factor that regulates the production of several detoxifying enzymes and antioxidants when it is translocated to the nucleus [43]. In this way, additional studies will be needed to evaluate DHCA potential in inhibiting NF-*κβ* and in activating the Nrf2 in fibroblasts exposed to UVB.

## 5. Conclusion

**Figure 8:**
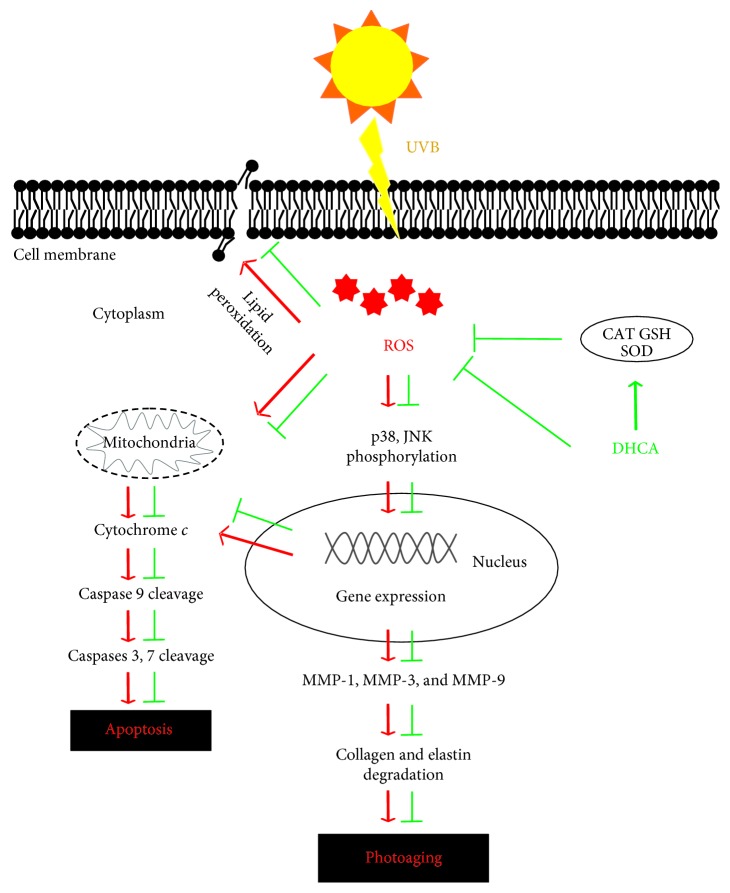
UVB-induced ROS production followed by the activation of both JNK and p38 MAPK subfamilies, leading to apoptosis and photoaging on fibroblasts (red arrows). The main DHCA protective effects against UVB on fibroblasts (green arrows).

We demonstrated for the first time that DHCA inhibits UVB-induced lipid peroxidation, apoptosis, and MMP-1 expression in L929 fibroblasts, thereby resulting in skin 1photoaging attenuation. These effects were associated with oxidative stress inhibition by decreasing ROS production and the regeneration of endogenous antioxidant defenses, which led to suppression of the p38 MAPK signaling pathway (Figure 8). Therefore, the incorporation of DHCA in topical delivery systems could modulate UVB-induced oxidative damage, consisting of a promising strategy to prevent skin photoaging.

## Figures and Tables

**Table 1:** Antioxidant capacity of DHCA and QT according to different cell-free antioxidant assays.

Samples	ABTS^•+^ (*μ*M TE/*μ*M)	DPPH^•^IC_50_ (*μ*M)	XOD IC_50_ (*μ*M)
DHCA	5.09 ± 0.76	7.65 ± 0.08^∗^	0.96 ± 0.03^∗^
QT	6.26 ± 0.23	8.48 ± 0.15	0.35 ± 0.02

Results are expressed as mean ± SD (*n* = 3). ^∗^ in each column indicates a significant difference compared with QT according to Tukey tests (*p* < 0.05). IC_50_: inhibitory concentration to 50%; TE: trolox equivalent.

## Data Availability

The authors declare that the data used to support the findings of this study are all included and available within the article.
